# Sirt6 ameliorates high glucose-induced podocyte cytoskeleton remodeling via the PI3K/AKT signaling pathway

**DOI:** 10.1080/0886022X.2024.2410396

**Published:** 2024-10-08

**Authors:** Zongwei Zhang, Hao Huang, Yu Tao, Hongyan Liu, Yanqin Fan

**Affiliations:** a Division of Nephrology, Renmin Hospital of Wuhan University, Wuhan, Hubei, China; b Division of Rehabilitation, Tianmen First People’s Hospital, Tianmen, Hubei, China; c Department of Physiology and Anatomy, University of North Texas Health Science Center, Fort Worth, USA

**Keywords:** Sirt6, diabetic kidney disease, podocyte injury, cytoskeletal remodeling, PI3K/AKT signaling pathway

## Abstract

**Background:**

Podocyte injury plays an important role in the occurrence and progression of diabetic kidney disease (DKD), which leads to albuminuria. Cytoskeletal remodeling is an early manifestation of podocyte injury in DKD. However, the underlying mechanism of cytoskeletal remodeling has not been clarified. Histone deacetylase sirtuin6 (Sirt6) has been found to play a key role in DKD progression, and the phosphatidylinositol 3-kinase (PI3K)/protein kinase B (PKB/AKT) pathway directly regulates the cytoskeletal structure of podocytes. Whereas, the relationship between Sirt6, the PI3K/AKT pathway and DKD progression remains unclear.

**Methods:**

Renal injury of db/db mice was observed by PAS staining and transmission electron microscope. Expression of Sirt6 in the glomeruli of db/db mice was detected by immunofluorescence. UBCS039, a Sirt6 activator, was used to explore the renal effects of Sirt6 activation on diabetic mouse kidneys. We also downregulating Sirt6 expression in podocytes using the Sirt6 inhibitor, OSS_128167, and induced upregulation of Sirt6 using a recombinant plasmid, after which the effects of Sirt6 on high glucose (HG)-induced podocyte damage were assessed *in vitro*. Podocyte cytoskeletal structures were observed by phalloidin staining. The podocyte apoptotic rate was assessed by flow cytometry, and PI3K/AKT signaling activation was measured by Western blotting.

**Results:**

Db/db mice exhibited renal damage including elevated urine albumin-to-creatinine ratio (ACR), increased mesangial matrix, fused podocyte foot processes, and thickened glomerular basement membrane. The expression of Sirt6 and PI3K/AKT pathway components was decreased in db/db mice. UBCS039 increased the expressions of Sirt6 and PI3K/AKT pathway components and ameliorated renal damage in db/db mice. We also observed consistent Sirt6 expression was in HG-induced podocytes *in vitro*. Activation of the PI3K/AKT pathway *via* a Sirt6 recombinant plasmid ameliorated podocyte cytoskeletal remodeling and apoptosis in HG-treated immortalized human podocytes *in vitro*, whereas Sirt6 inhibition by OSS_128167 accelerated HG-induced podocyte damage *in vitro*.

**Conclusions:**

Sirt6 protects podocytes against HG-induced cytoskeletal remodeling and apoptosis through activation of the PI3K/AKT signaling pathway. These findings provide evidence supporting the potential efficacy of Sirt6 activation as a promising therapeutic strategy for addressing podocyte injury in DKD.

## Introduction

Diabetic kidney disease (DKD) is one of the most severe microvascular complications of diabetes and is the main risk factor for progression to end-stage renal disease (ESRD) in patients with renal diseases [1]. Although one-third of diabetic patients develop DKD [2]. There are no curative therapies available; therefore, there is an urgent unmet need to identify effective treatments for patients with DKD.

Proteinuria is an early manifestation of DKD in most patients. Podocytes foot processes extending from the cell body wrap around the outside of the glomerular basement membrane (GBM), and podocyte injury is the primary reason for albuminuria in DKD [3–5]. In a diabetic state, podocyte cytoskeletal remodeling caused by hyperglycemia leads to cell apoptosis and contributes to the pathogenesis of DKD [6]. Furthermore, ameliorating podocyte cytoskeletal remodeling can alleviate both podocyte injury and DKD progression [7–9]. Thus, identifying the pivotal molecules involved in podocyte cytoskeletal remodeling could provide valuable insights to aid the development of novel therapeutic strategies for patients with DKD.

As a member of the sirtuins family, Sirt6 functions as an NAD^+^-dependent deacetylase and an ADP-ribonyltransferase [10]. Sirt6 is implicated in a variety of biological processes including DNA repair, glucose/lipid metabolism and transcriptional regulation [10,11] and is also associated with the development of multiple kidney diseases, including DKD [12]. Overexpression of Sirt6 can promote M2 macrophage transformation, which alleviates renal injury in diabetic nephro­pathy [13]. Another study found that Sirt6 could restrain mitochondrial malfunction and apoptosis in HG-treated podocytes *via* adenosine monophosphate-activated protein kinase (AMPK) signaling [14]. Besides, Sirt6 overexpression was shown to reduce HG-induced podocyte cytoskeletal damage [15]. Miao et al. found that Sirt6 suppressed podocyte injury by inhibiting RAS signaling by the Wnt1/β- catenin pathway in rats of membranous nephropathy [16]. Importantly, the mechanisms underlying the role of Sirt6 in the regulation of podocyte cytoskeletal structures need to be further elucidated.

Phosphatidylinositol 3-kinase (PI3K)/protein kinase B (PKB/AKT) signaling pathway is one of the core signaling pathways that regulate cell growth, proliferation, motility, metabolism and survival [17]. Moreover, data have shown that the PI3K/AKT pathway directly regulates the cytoskeletal structure of podocytes [18]. Hyperglycemia can lead to the suppression of PI3K/AKT pathway activity [19], and failure to phosphorylate Akt in podocytes from db/db mice with early DKD has been shown to result in cell death [20]. Intriguingly, studies have found that Sirt6 can regulate the PI3K/AKT pathway in cancer [21,22]. Thus, we propose that Sirt6 might restrain HG-induced cytoskeletal remodeling and podocyte damage *via* PI3K/AKT pathway activation.

In the present study, we explored the mechanisms underlying the role of Sirt6 in HG-insulted podocyte cytoskeletal remodeling and injury.

## Results

### Renal injury of db/db mice

**Figure 1.**
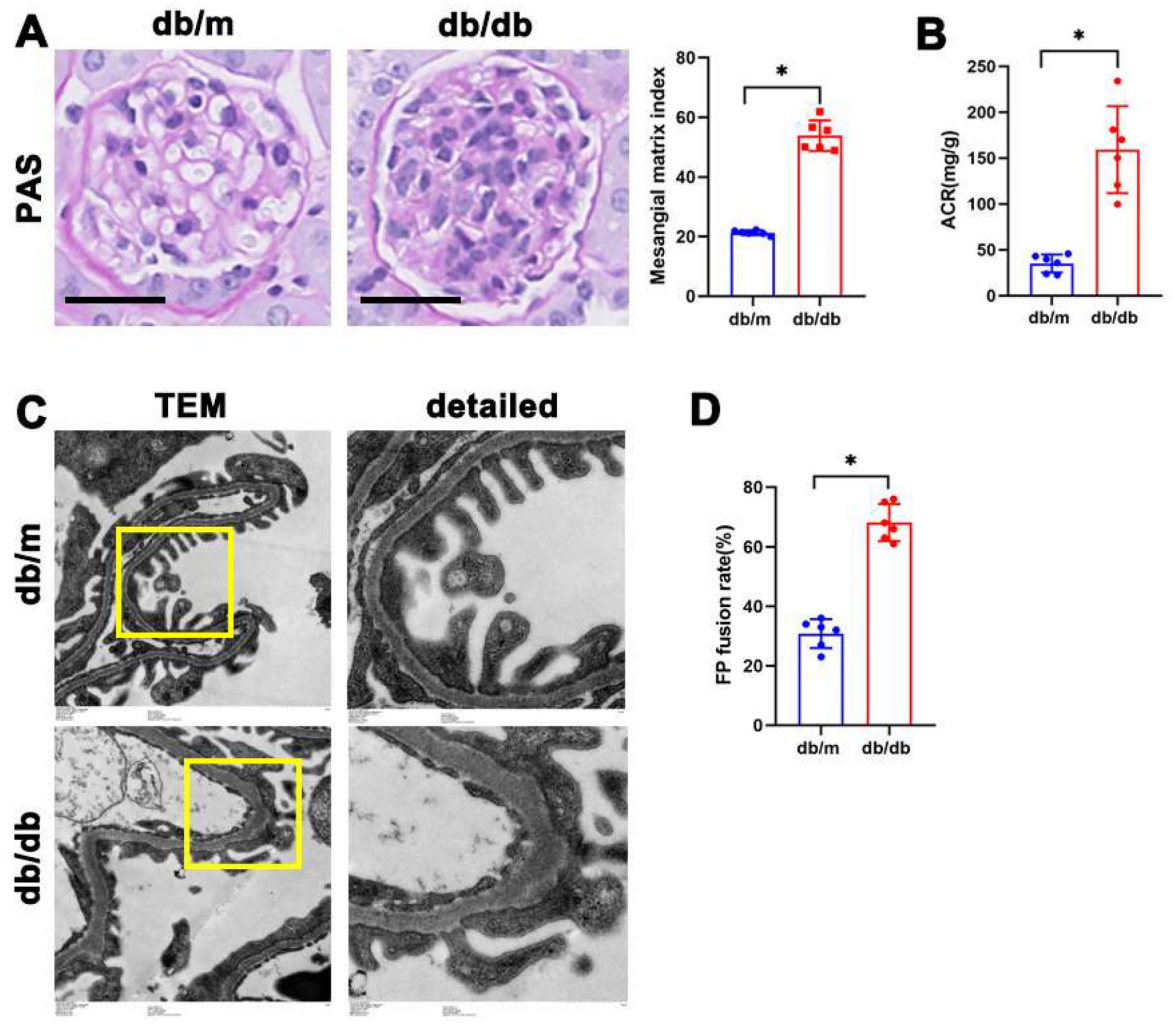
Renal injury in db/db mice. (A) Representative microscopy images and quantification of PAS staining of the glomeruli of kidneys from db/m and db/db mice (original magnification ×400). (B) Quantitative analysis of ACR (albumin-to-creatinine ratio) in db/m and db/db mice. (C) Representative transmission electron microscopy images of the ultrastructure of capillary loops in each group (original magnification ×8,000, ×12,000). (D) Foot process fusion rate in db/m and db/db mice. n = 6. *P < 0.05. Scale bars: 20 µm.

Periodic acid Schiff (PAS) staining, transmission electron microscopy, and ACR measurement were conducted to investigate alterations in glomerular and podocyte damage in 24-week-old db/db mice. Consistent with other findings [23], PAS staining demonstrated a dilated mesangial matrix in the glomeruli of kidney from db/db mice (Figure 1(A)). ACR values were higher in db/db mice compared to the control group (Figure 1(B)). Electron microscopy showed fused podocyte foot processes and thickened glomerular basement membrane in db/db mice (Figure 1(C and D)). All the results indicate glomeruli and podocyte damage in db/db mice.

### Sirt6 and PI3K/AKT expression in glomeruli of kidneys from db/db mice

**Figure 2.**
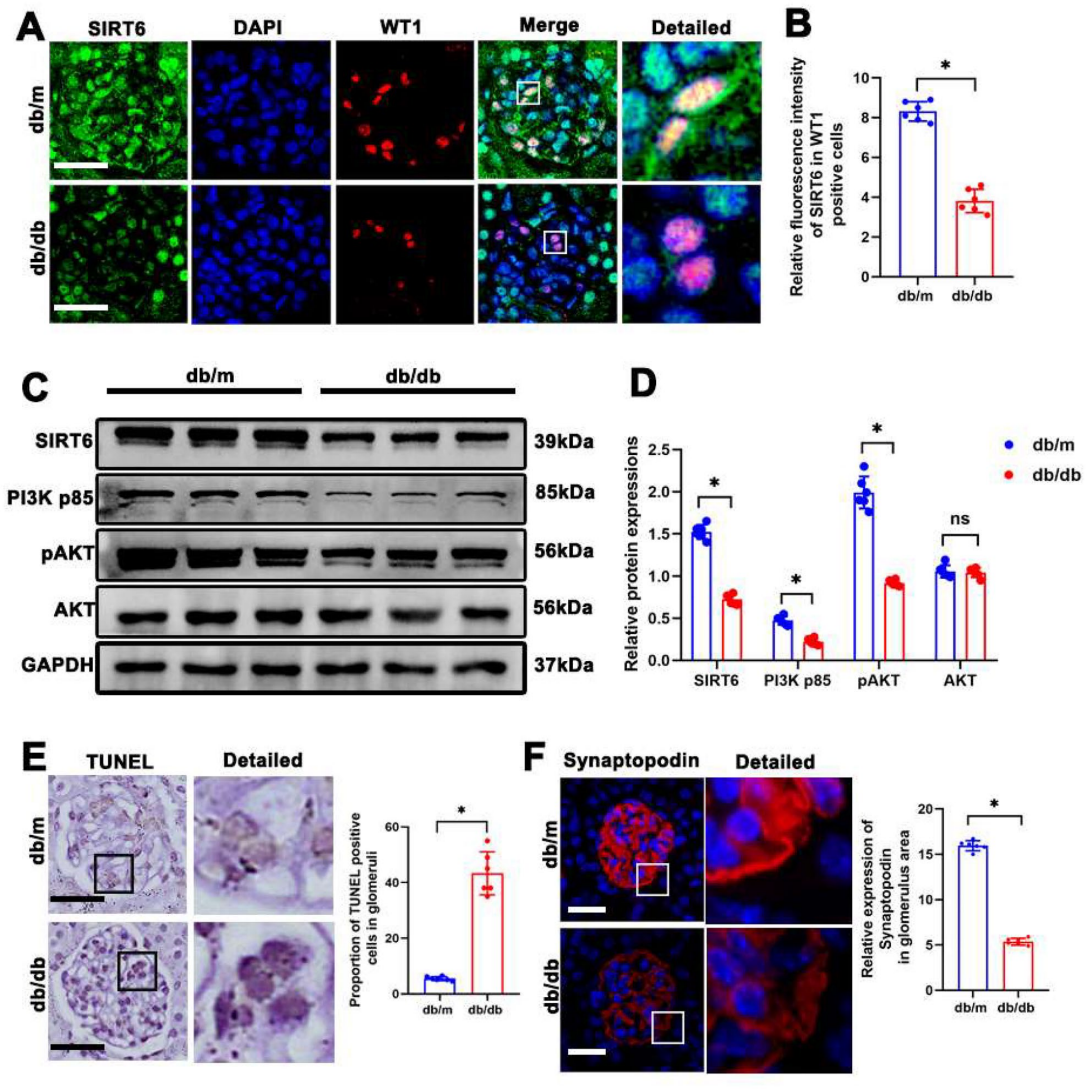
Sirt6 And PI3K/AKT pathway was downregulated in podocytes from db/db mice. (A and B) Representative immunofluorescent staining of Sirt6 in podocytes from db/m and db/db mice. WT-1 was used as a podocyte marker (original magnification ×400). (C and D) Western blotting analyses of Sirt6, PI3K p85, pAKT, and AKT expression in kidneys from db/m and db/db mice. GAPDH was set as loading control. (E) Representative immunohistochemistry showing TUNEL staining of glomeruli from db/m and db/db mice (original magnification ×400). (F) Representative immunofluorescent staining of glomerular Synaptopodin in db/m and db/db mice (original magnification ×400). *n* = 6. NS = not significant; *P < 0.05. Scale bars: 20 µm. PI3K p85: phosphoinositide 3-kinase subunit 85; AKT: protein kinase B; pAKT: phosphorylated Akt (Ser 473).

We first explored Sirt6 protein levels in podocytes of db/db mice by double immunofluorescent staining of Sirt6 and the podocyte marker, wilms tumor protein (WT1) (Figure 2(A and B)). Consistent with previous studies [14,15], Sirt6 expression was decreased in podocytes of db/db mice. In addition, Western blotting consistently showed that Sirt6 expression was reduced in the kidneys of db/db mice (Figure 2(C and D)). We also observed decreased abundance of activated PI3K and AKT in db/db mice, which indicated inhibition of the PI3K/AKT pathway (Figure 2(C and D)). Since podocyte apoptosis is a critical factor for the development of DKD, we used terminal-deoxynucleoitidyl transferase mediated nick end labeling (TUNEL) staining to shown that more apoptotic cells were observed in the glomeruli of db/db mice (Figure 2(E)). Synaptopodin is crucial for maintaining the normal function of the podocyte cytoskeletal structure. Using immunofluorescent staining, we found that Synaptopodin expression was decreased in the glomeruli of db/db mice (Figure 2(F)), which indicated dysfunction of the podocyte cytoskeleton.

### Sirt6 activation reduced renal damage in db/db mice

**Figure 3.**
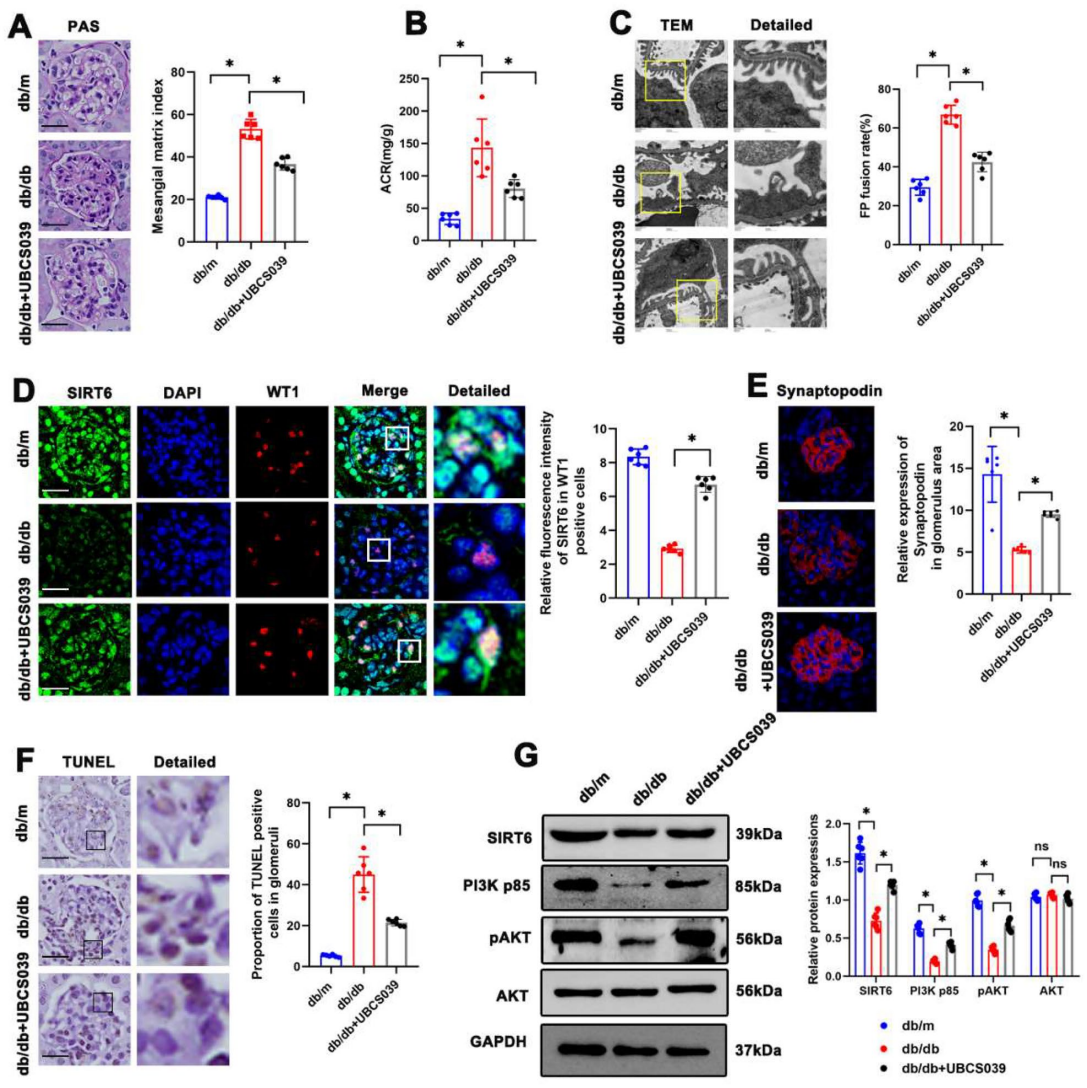
Sirt6 activation attenuated renal injury in db/db mice. (A) Representative microscopy images and quantification of PAS staining of the glomeruli in each group (original magnification ×400). (B) Quantitative analysis of ACR in each group. (C) Representative transmission electron microscopy images of the ultrastructure of capillary loops and foot process fusion rate in each group (original magnification ×8,000, ×12,000). (D) Representative immunofluorescent staining of Sirt6 expression in podocytes each group. WT-1 was used as a podocyte marker (original magnification ×400). (E) Representative immunofluorescent staining of glomerular Synaptopodin in each group (original magnification ×400). (F) Representative immunohistochemistry showing TUNEL staining of glomeruli in each group (original magnification ×400). (G) Western blotting analyses of Sirt6, PI3K p85, pAKT, and AKT expression in kidneys in each group. GAPDH was set as loading control. n = 6. NS = not significant; *P < 0.05. Scale bars: 20 µm. PI3K p85: phosphoinositide 3-kinase subunit 85; AKT: protein kinase B; pAKT: phosphorylated Akt (Ser 473).

To further probe into the role of Sirt6 in podocytes from db/db mice, UBCS039, an agonist of Sirt6 [24], was administrated. PAS staining indicated that UBCS039 ameliorated the mesangial matrix expansion in the glomeruli of db/db mice (Figure 3(A)). Moreover, urine ACR levels were significantly decreased in db/db mice following treatment with UBCS039 (Figure 3(B)). Consistently, electron microscopy revealed that fused podocyte foot processes and thickened glomerular basement membrane in db/db mice were alleviated by UBCS039 treatment (Figure 3(C)). We also observed the UBCS039-induced activation of Sirt6 (Figure 3(D)). Immunofluorescent staining of Synaptopodin showed that Sirt6 activation increased the expression of Synaptopodin in the glomeruli of db/db mice (Figure 3(E)). TUNEL staining showed that UBCS039 reduced the apoptotic rate of cells in the glomeruli of db/db mice (Figure 3(F)). As described above, PI3K/AKT pathway activity was decreased in db/db mice. However, Western blotting showed that UBCS039 partially reactivated the PI3K/AKT pathway in the kidneys of db/db mice (Figure 3(G)). We also explored the effects of UBCS039 on db/m mice. It suggested that there were no significant differences in PAS and TUNEL results between UBCS039 + db/m and db/m groups (Supplementary Fig. 1A).

### Effects of HG on Sirt6 expression, PI3K/AKT pathway activation, and cytoskeletal structures in cultured podocytes

**Figure 4.**
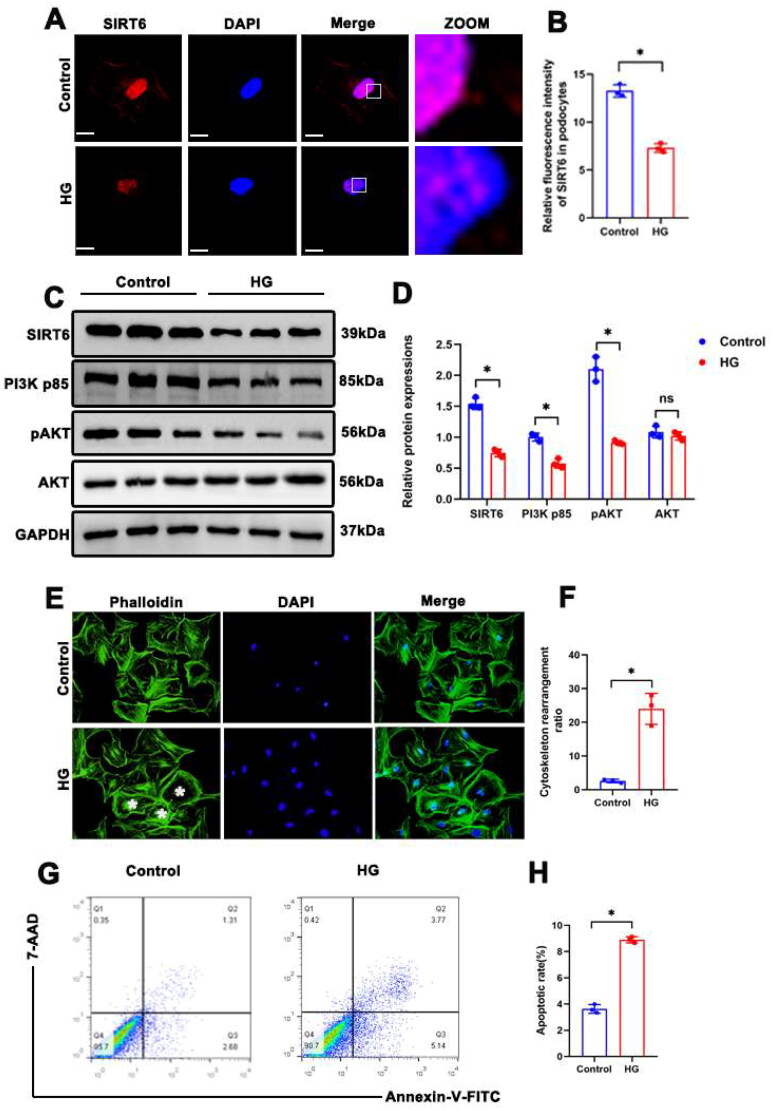
Effects of HG on Sirt6 expression, PI3K/AKT pathway activation and cytoskeletal structure in cultured podocytes. (A and B) Representative immunofluorescent staining of Sirt6 expression in podocytes in each group (original magnification ×1000). (C and D) Western blotting analyses of Sirt6, PI3K p85, pAKT, and AKT expression in each group. GAPDH was set as loading control. (E and F) Cytoskeleton structure of podocytes labeled with phalloidin. * Podocyte with cytoskeletal remodeling (original magnification ×1000). (G and H) Flow cytometry analysis of apoptosis in cultured podocytes in different groups and quantitation of these results. *n* = 3. NS = not significant; *P < 0.05. Scale bars: 10 µm. PI3K p85: phosphoinositide 3-kinase subunit 85; AKT: protein kinase B; pAKT: phosphorylated Akt (Ser 473); HG: high glucose (30 mM).

A previous study showed that the expression of Sirt6 and PI3K/AKT pathway components was downregulated in podocytes of diabetic mice *in vivo* [14,25]. We found that HG stimulation induced a decreased in Sirt6 expression in cultured podocytes. As shown in Figure 4(A and B), immunofluorescent staining showed that Sirt6 was reduced in HG-stimulated podocytes. Western blotting also showed decreased Sirt6 expression and PI3K/AKT pathway inhibition in podocytes in HG-treated group (Figure 4(C and D)). Importantly, phalloidin staining showed F-actin rearrangement in HG-treated podocytes (Figure 4(E and F)), which suggested podocyte cytoskeletal remodeling. Moreover, we also showed that HG caused increased podocyte apoptotic rate (Figure 4(G and H)), which was similar to other study [14,26].

### Inhibition of Sirt6 Aggravated HG-induced cytoskeletal remodeling in podocytes *in vitro*

**Figure 5.**
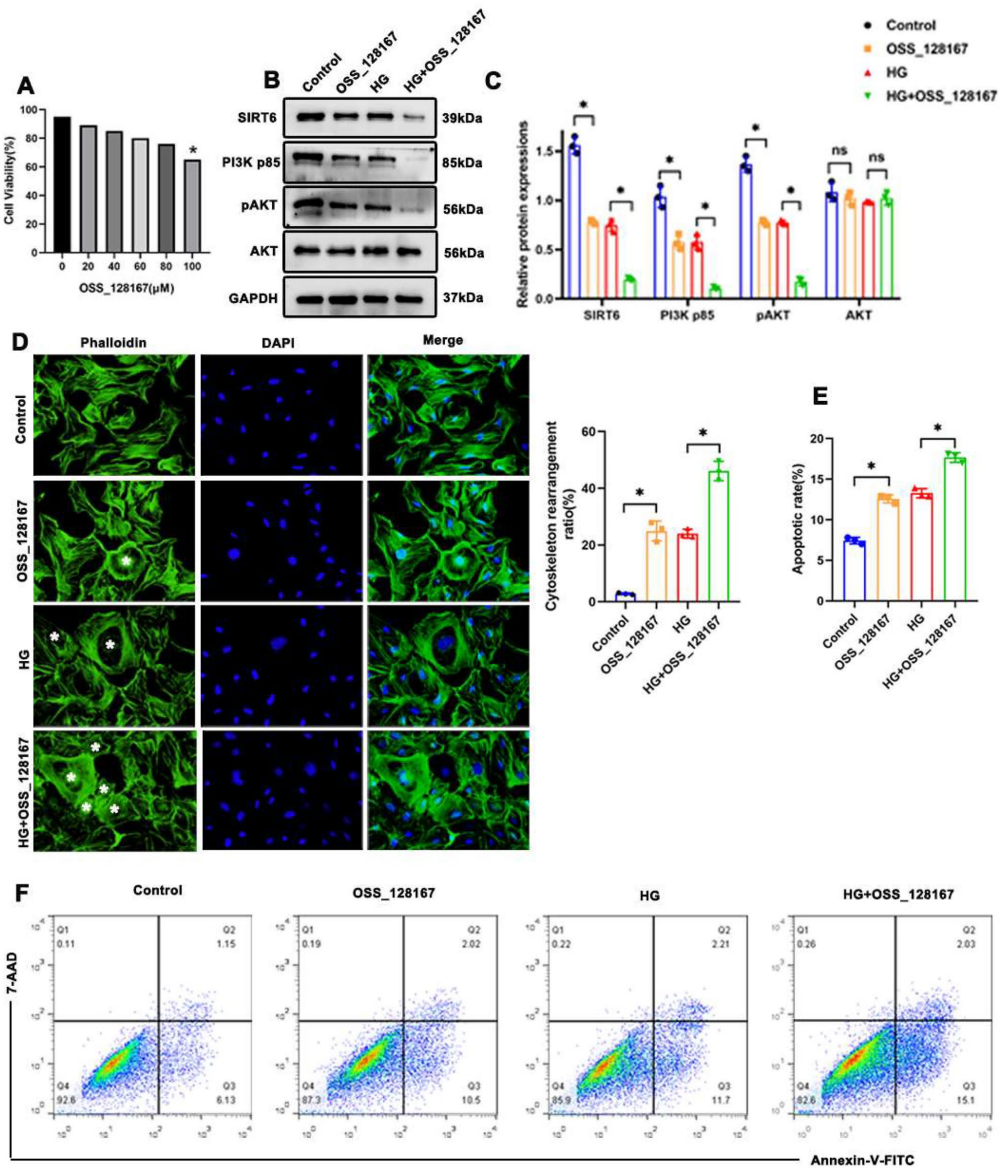
Inhibition of Sirt6 Aggravated HG-induced cytoskeletal remodeling in podocytes *in vitro*. (A) CCK-8 assay showing cell viability following treatment with OSS_128167. (B and C) Western blotting analyses of Sirt6, PI3K p85, pAKT, and AKT expression in each group. GAPDH was set as loading control. (D) Cytoskeleton structure of podocytes labeled with phalloidin (original magnification ×1000). * Podocyte with cytoskeletal remodeling. (E and F) Flow cytometry analysis of apoptosis in cultured podocytes in different groups and quantitation. *n* = 3. NS = not significant; *P < 0.05. PI3K p85: phosphoinositide 3-kinase subunit 85; AKT: protein kinase B; pAKT: phosphorylated Akt (Ser 473); HG: high glucose (30 mM).

*In vitro* studies have shown that both Sirt6 and PI3K/AKT pathway activation are decreased in HG-stimulated podocytes. However, whether Sirt6 alteration is connected with the PI3K/AKT signaling pathway, and the precise relationship between Sirt6, the PI3K/AKT pathway, and podocyte injury remain unknown. We treated podocytes with OSS_128167, an Sirt6 inhibitor [21], and measured viability using a cell counting kit (CCK)-8 assay. As shown in Figure 5(A), cell viability was about 60% at 100 μM dose. Western blotting suggested that OSS_128167 reduced Sirt6 protein expression and decreased activation of the PI3K/AKT pathway in podocytes (Figure 5(B and C)). Besides, OSS_128167 treatment exacerbated HG-induced cytoskeletal remodeling in podocytes (Figure 5(D)) and increased the podocyte apoptotic rate (Figure 5(E and F)). These results suggested that Sirt6 inhibition exacerbated cytoskeletal remodeling and apoptosis in HG-treated podocytes by suppressing of PI3K/AKT signaling pathway.

### Sirt6 overexpression ameliorated HG-induced podocyte cytoskeletal remodeling and apoptosis

**Figure 6.**
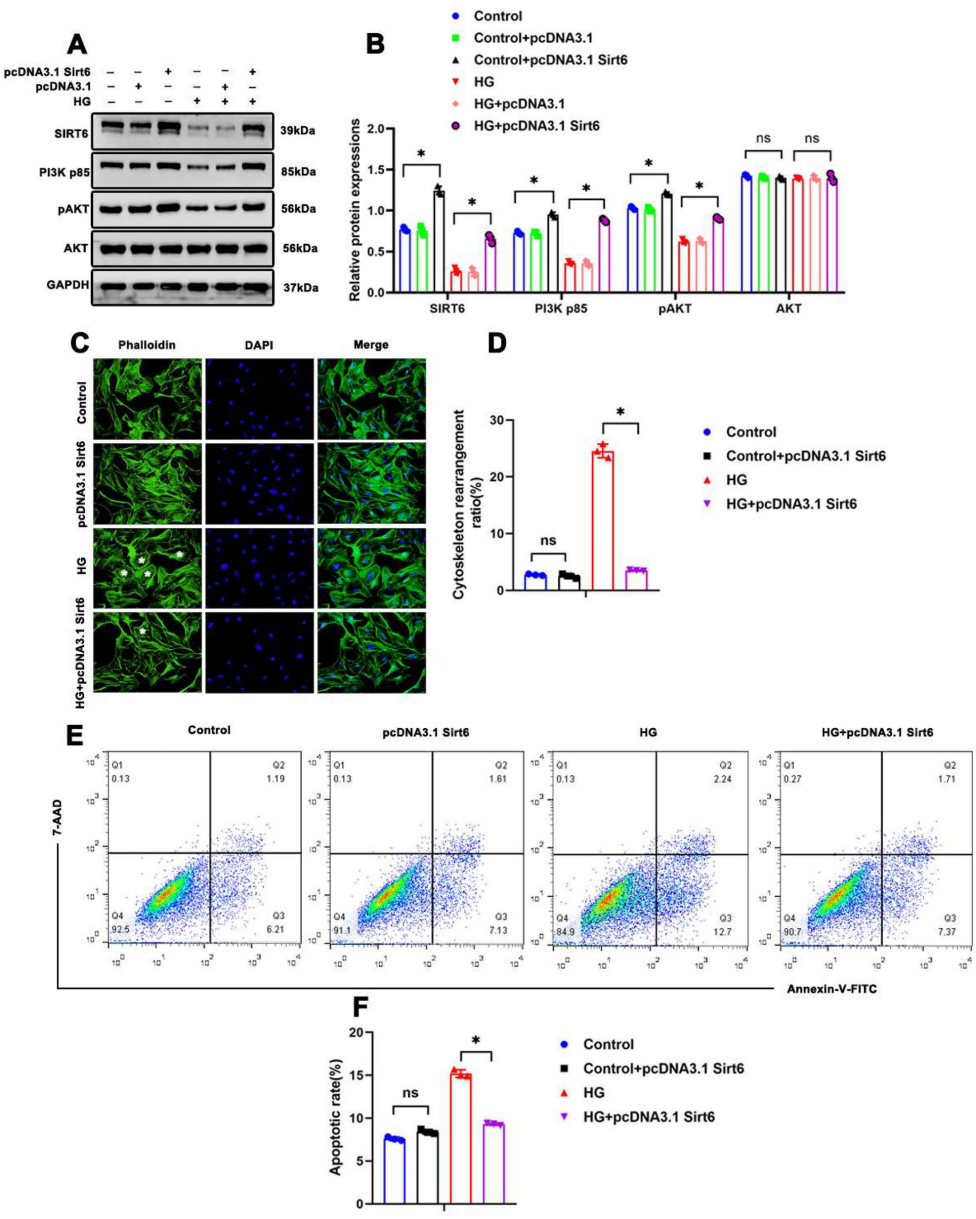
Sirt6 overexpression ameliorated HG-induced podocyte cytoskeletal remodeling and apoptosis. (A and B) Western blotting analyses of Sirt6, PI3K p85, pAKT, and AKT expression in each group. GAPDH was set as loading control. (C and D) Cytoskeleton structure of podocytes labeled with phalloidin (original magnification ×1000). * Podocyte with cytoskeletal remodeling. (E and F) Flow cytometry analysis of apoptosis in cultured podocytes in different groups and quantitation. *n* = 3. NS = not significant; *P < 0.05. PI3K p85: phosphoinositide 3-kinase subunit 85; AKT: protein kinase B; pAKT: phosphorylated Akt (Ser 473); HG: high glucose (30 mM).

In order to explore the effect of Sirt6 overexpression on podocytes *in vitro*, a recombinant plasmid (pcDNA3.1 Sirt6) was transfected into cultured podocytes. Western blotting showed that Sirt6 overexpression partially restored the expression of the activated PI3K/AKT pathway in HG-treated podocytes (Figure 6(A and B)). Moreover, Sirt6 overexpression ameliorated the cytoskeletal remodeling and apoptosis in HG-treated podocytes (Figure 6(C–F)). Hence, Sirt6 can protect podocytes from HG-induced damage by activating of PI3K/AKT signaling pathway.

## Discussion

Cytoskeletal remodeling plays a vital role in hyperglycemia-induced podocyte damage and is implicated in DKD progression [27,28]. In this study, we detected that Sirt6 expression was reduced in db/db diabetic mice *in vivo* and that HG-stimulated podocyte cytoskeleton remodeling *in vitro*. Activation of Sirt6 can ameliorate podocyte injury and proteinuria in db/db mice and overexpression of Sirt6 can reduce cytoskeletal remodeling and apoptosis in HG-stimulated podocytes. Mechanistically, we showed that Sirt6 protected podocytes from HG-induced injury by activating the PI3K/AKT signaling pathway.

Sirt6, an important member of the sirtuins family, has been shown to mediate a variety of physiological processes in the kidney [12]. As an upstream nucleoprotein, Sirt6 can regulate cellular homeostasis *via* multiple biological processes, including telomere maintenance, DNA repair, energy metabolism, oxidative stress, the inflammatory response, and fibrosis [10]. Previous study has found that loss of Sirt6 accelerates podocyte damage, renal hypertrophy and progressive proteinuria [29]. In addition, Sirt6 has been suggested to prevent the DKD progression [14]. We previously showed that Sirt6 overexpression can ameliorate mitochondrial malfunction and suppress oxidative stress by stimulating AMPK phosphorylation following HG-induced podocyte injury [14]. Besides, Sirt6 can regulate H3K9 deacetylation and bind to the promoter regions of Notch1 and Notch4 to inhibit phosphatase and tensin homolog (PTEN) transcription and activation. This promotes autophagy, inhibits apoptosis and reduces the inflammatory response in podocytes [15]. Moreover, Sirt6 was reported to play a protective role in podocytes in DKD by accelerating M2 macrophage transformation, and as an immune regulator in inflammation damage [13]. Yang et al. found that Sirt6 was a potential target for renin-angiotensin system ­(RAS)-associated podocyte injury[30]. Furthermore, proximal tubule NMN-producing enzyme nicotinamide phosphoribosyl-transferase (Nampt)-specific knockout mice with Sirt6 downregulation exhibited collagen deposition and a fibrotic phenotype, suggesting that Sirt6 is involved in renal fibrosis in DKD [31]. In our study, Sirt6 had a protective effect on podocytes following HG-induced cytoskeletal remodeling and apoptosis by activating of PI3K/AKT signaling pathway, which further confirmed the protective role of Sirt6 in DKD.

Podocytes exhibit a unique cytoskeletal architecture fundamentally linked to their function in maintaining the kidney filtration barrier, which regulates podocyte shape, structure, stability, slit diaphragm insertion, adhesion, plasticity, and dynamic response to environmental stimulations. Thus, even slight impairment of the podocyte cytoskeletal apparatus can result in proteinuria and glomerular disease [32]. Podocyte cytoskeletal structure abnormalities have been widely reported in diabetic conditions. However, the underlying mechanism of cytoskeletal damage remains unclear. Synaptopodin is a proline-rich, actin-binding protein distributed in the peduncle and neuronal dendritic spines of podocytes. Studies have reported that under physiological conditions, knockdown of Synaptopodin did not cause a significant renal phenotype, and there was no significant difference in urinary protein levels between control and Synaptopodin knockout mice. However, under pathological conditions, Synaptopodin knockdown exacerbated glomerular podocyte injury in adriamycin nephropathy glomeruli, with Synaptopodin knockdown mice showing more severe urinary protein, hematuria and glomerulosclerosis compared to controls [33]. In addition, studies using primary cultured podocytes showed that Synaptopodin deficiency resulted in loss of stress fibers and impaired cell migration ability [33]. Another study showed that Synaptopodin deficiency exacerbated the pathological damage in a mouse model of Alport syndrome by aggravating the abnormal glomerular basement membrane structure and loss of podocytes [34]. Here, we found that Synaptopodin expression was significantly decreased in the glomeruli of db/db mice. Moreover, Sirt6 activation by UBCS039 increased Synaptopodin expression and ameliorated proteinuria and podocyte foot process fusion in db/db mice.

Physiologically, insulin stimulates the PI3K pathway and leads to AKT phosphorylation and activation. AKT exists in three isoforms, Akt1 (PKBα), Akt2 (PKBβ) and Akt3 (PKBγ), among which AKT2 is located specifically in the podocyte and is the main isoform through which insulin signals [35]. PI3K and AKT are key insulin signaling molecules, which have significant regulatory effects on glucose and lipid metabolism. After activation, pAKT can regulate glucose transporter-4 (GLUT-4), GSK-3β, forkhead box protein O1 (FoxO1), mTOR and other effector molecules to regulate glucose and lipid metabolism, cell autophagy and apoptosis, inflammation, oxidative stress levels, thus participating in the development of DKD [36]. The loss of podocyte AKT2 activation is detrimental in DKD [35]. Studies have found that the PI3K/AKT pathway is closely involved in the regulation of podocyte cytoskeletal structure [19]. In our study, we observed recovered podocyte cytoskeletal remodeling when the PI3K/AKT pathway was activated. Furthermore, Sirt6 overexpression alleviated podocyte injury both *in vitro* and *in vivo* by activating the PI3K/AKT pathway.

One limitation of our study is that the molecular mechanisms underlying Sirt6-induced activation of the PI3K/AKT were not elucidated. Sirt6 was reported to regulate the PI3K/AKT pathway in several disease states. For instance, Sirt6 can promote tumorigenesis and drug resistance of diffuse large B-cell lymphoma through PI3K/Akt signaling [21]. Sirt6 also functions as a tumor suppressor gene in colon cancer by regulating the PTEN/AKT pathway [37]. In addition, Sirt6 can promote the radiosensitivity in non-small cell lung cancer (NSCLC) and inhibit the development of NSCLC by suppressing the activity of the PI3K/Akt/mTOR signaling pathway [22]. Consistently, our results showed that the upregulation of Sirt6 led to the activation of the PI3K/AKT signaling pathway and suppression of Sirt6 inhibited the PI3K/AKT pathway. The interaction between Sirt6 and the PI3K/AKT pathway in podocytes should be further explored in future studies.

In conclusion, our results indicate that Sirt6 protects podocytes against HG-induced damage in diabetic mice by inhibiting podocyte cytoskeletal remodeling and apoptosis through activation of the PI3K/AKT pathway. Thus, upregulated Sirt6 might be a potential therapeutic target for DKD; however, future studies are needed to confirm the feasibility of Sirt6 as a therapeutic target in patients with DKD.

### Materials and methods

#### Reagents

UBCS039 (HY-115453) and OSS_128167 (HY-107454) were obtained from MedChemExpress (Shanghai, China). Sirt6 primary antibody (13572–1-AP) was purchased from ProteinTech (Wuhan, China). Akt, Phospho-Akt (Ser473) (# 9271), and PI3K p85 (# 4257) antibodies were purchased from Cell Signaling Technology (USA). Anti-GAPDH (sc − 47724), WT1 (sc − 7385) and Synaptopodin (sc − 515842) antibodies were purchased from Santa Cruz (USA). DAPI and secondary antibodies against rabbit IgG-HRP, IgG-HRP, Alexa Flour 488, 594 conjugated anti-mouse IgG, anti-rabbit IgG and were obtained from Antgene (Wuhan, China).

#### Animal studies

Db/m and db/db male mice (24 weeks, 30–50 g) were obtained from CAWENS animal company (Changzhou, China) and were raised in specific pathogen-free conditions at the Center for Animal Experiments of Renmin Hospital of Wuhan University. All protocols were approved by the Animal Ethics Review Board of Renmin Hospital of Wuhan University in advance of the experiments (ethical approval number: 20200306). The experiments were executed according to the guidelines of the National Health and Medical Research Council of China (*n* = 6 each): db/m, db/db and db/db plus UBCS039. In the db/db plus UBCS039 group, mice were given UBCS039 (50 mg/kg) by intraperitoneal injection for two weeks. We collected their 24-h urine and measured urine albumin-to-creatinine ratio (ACR). The animals were sacrificed, and their kidneys were perfused with physiological saline and stored at −80 °C. A portion of the kidney tissues were fixed with glutaraldehyde prior to electron microscope examination.

#### Histology and immunohistochemistry (IHC) examination

Kidney tissues were fixed in 4% paraformaldehyde (pH 7.4) for 24 h. The tissues were embedded in paraffin, sectioned, stained with periodic acid-schiff stain (PAS), then observed with the microscope (Olympus, Tokyo, Japan). For IHC staining, sections were incubated with indicated primary antibodies. Overall, six visual fields from individual groups were randomly selected to calculate the percentage of positive staining area using Image J. The individuals who evaluated the results of histology and IHC staining were masked to sample identity.

#### Cell culture

Human podocytes were originally provided by Dr Saleem MA from the University of Bristol, UK. The medium consisted of RPMI 1640 (HyClone, USA) containing 10% heat-inactivated fetal bovine serum (FBS; Gibco, USA), 100 U/mL penicillin G, 100 μg/mL streptomycin (Invitrogen, USA) and insulin-transferrin-selenium (ITS; Invitrogen, USA) at 33 °C. To induce differentiation, podocytes were cultured at 37 °C without ITS, and the differentiated podocytes were used in the following experiments. The differentiated podocytes were treated with 30 mM high glucose (HG) for 24 h.

In order to inhibit Sirt6 expression, OSS_128167 (100 μM) was added to complete medium. To upregulate Sirt6 expression, the Sirt6 plasmid (Miaoling, China) was transfected into podocytes using the X-tremeGENE HP DNA Transfection Reagent (Roche). Then, the cells were incubated in complete medium for 24 h at 37 °C, and stimulated with 30 mM HG as necessary.

#### Western blotting

After treatment, the samples were lysated in RIPA lysis buffer with PMSF and protease inhibitor cocktail for 30 min at 4 °C. The proteins were loaded on to 10% SDS-PAGE gel and transferred onto PVDF membrane. Next, 5% milk was used to block the PVDF membrane for 1 h. Then, membranes were incubated with primary antibodies (Sirt6 rabbit monoclonal antibody, 1:1000, ProteinTech; Akt rabbit monoclonal antibody, 1:1000, CST; p-Akt rabbit monoclonal antibody, 1:1000, CST; PI3K p85 rabbit monoclonal antibody, 1:1000, CST; GAPDH mouse monoclonal antibody, 1:1000, ProteinTech) overnight at 4 °C. Then incubated the bands with secondary antibody (Antgene, China). The bands were visualized using ECL chemiluminescent kit (Servicebio, China) and analyzed by ChemiDocTM MP Imaging system (Bio-Rad, USA).

#### Cytoskeleton staining

Phalloidin (Abcam, USA) was used to stain the podocyte cytoskeleton. After treatment, podocytes were washed with PBS, fixed in 4% paraformaldehyde, and blocked with 5% bovine serum albumin (BSA), then stained with phalloidin for 60 min. The nuclei were stained with DAPI. Slides were then viewed under a fluorescence microscope. Under normal conditions, F-actin presents a clear uniform bundle distribution across cells along the podocyte axis; cytoskeleton remodeling is characterized by accumulation of F-actin in the peri-cellular area, arranged along the periphery of the cell, and slightly diffused cytoplasmic distribution. The individuals who evaluated the results of cytoskeleton remodeling were masked to sample identity.

#### Immunofluorescence staining

Fixing the podocytes with 4% paraformaldehyde and blocking cells with 5% BSA after treatment. Incubating the cells with specific primary antibodies (Sirt6 rabbit monoclonal antibody, 1:1000, proteintech) overnight at 4 °C. Next, the samples were incubated with fluorescent secondary antibodies for 1h. After washing, the nuclei were counterstained with DAPI. Fluorescence results were analyzed using confocal laser microscope (Olympus, Japan). The individuals who evaluated the results of IF staining were masked to sample identity.

#### Apoptosis assay

The degree of podocyte apoptosis *in vitro* was assessed by flow cytometry with Annexin V-FITC/7-AAD double staining according to the manufacturer’s instructions (BD Pharmingen, USA). Podocyte apoptosis in kidney tissues was detected by IHC staining with TUNEL following the reference instructions (Roche Applied Science, Germany).

#### CCK-8 experiment

Cell viability of podocytes was performed by cell counting kit- 8 assay according to reference instructions (Dojindo, Japan). Briefly, podocytes were grown in 96-well plates at a density of 1 × 10^4^ cells/well, then treated with different concentrations of OSS_128167 for 24 h. Culture medium was replaced by 10 μL CCK-8 dye and 90 μl medium for 1 h at 37 °C after the above stimulation. Content determination of formazan dye produced by cellular dehydrogenase activity in podocytes was observed at 450 nm wavelength.

#### Statistical analysis

Statistical analysis was performed using SPSS 20.0 software. Bar graphs were generated by Graphpad Prism (GraphPad, La Jolla, CA). The data were represented as mean ± standard deviation. The statistical difference between two groups was calculated by Student’s *t* test. Statistical data among multiple groups were analyzed by one‑way ANOVA. The statistical differences were considered significant when *p* < 0.05.

## Supplementary Material

Supplemental Material
