# Supplementary material for: Sirt6 ameliorates high glucose-induced podocyte cytoskeleton remodeling via the PI3K/AKT signaling pathway
Source: Ren Fail. 2024 Oct 8;46(2):2410396. doi: 10.1080/0886022X.2024.2410396 (PMC11463017; doi:10.1080/0886022X.2024.2410396)

**ONLINE SUPPLEMENT**

**Materials and methods**

**Animal studies**

Db/m male mice (24 weeks, 30-50g) were obtained from CAWENS animal company (Changzhou, China) and were raised in specific pathogen-free conditions at the Center for Animal Experiments of Renmin Hospital of Wuhan University. All protocols were approved by the Animal Ethics Review Board of Renmin Hospital of Wuhan University. The experiments were executed according to the guidelines of the National Health and Medical Research Council of China (n = 6 each): db/m, and db/m plus UBCS039. In the db/m plus UBCS039 group, mice were given UBCS039 (50 mg/kg) by intraperitoneal injection for two weeks. The animals were sacrificed, and part of kidneys was fixed in 4% phosphate-buffered paraformaldehyde for pathological analysis

**Supplementary Figure 1. Effects of UBCS039 on db/m mice**

1. Representative microscopy images and quantification of PAS staining of the glomeruli in each group (original magnification ×400). (B) Representative immunohistochemistry showing TUNEL staining of glomeruli in each group. n = 6. NS = not significant. Scale bars: 25 µm.


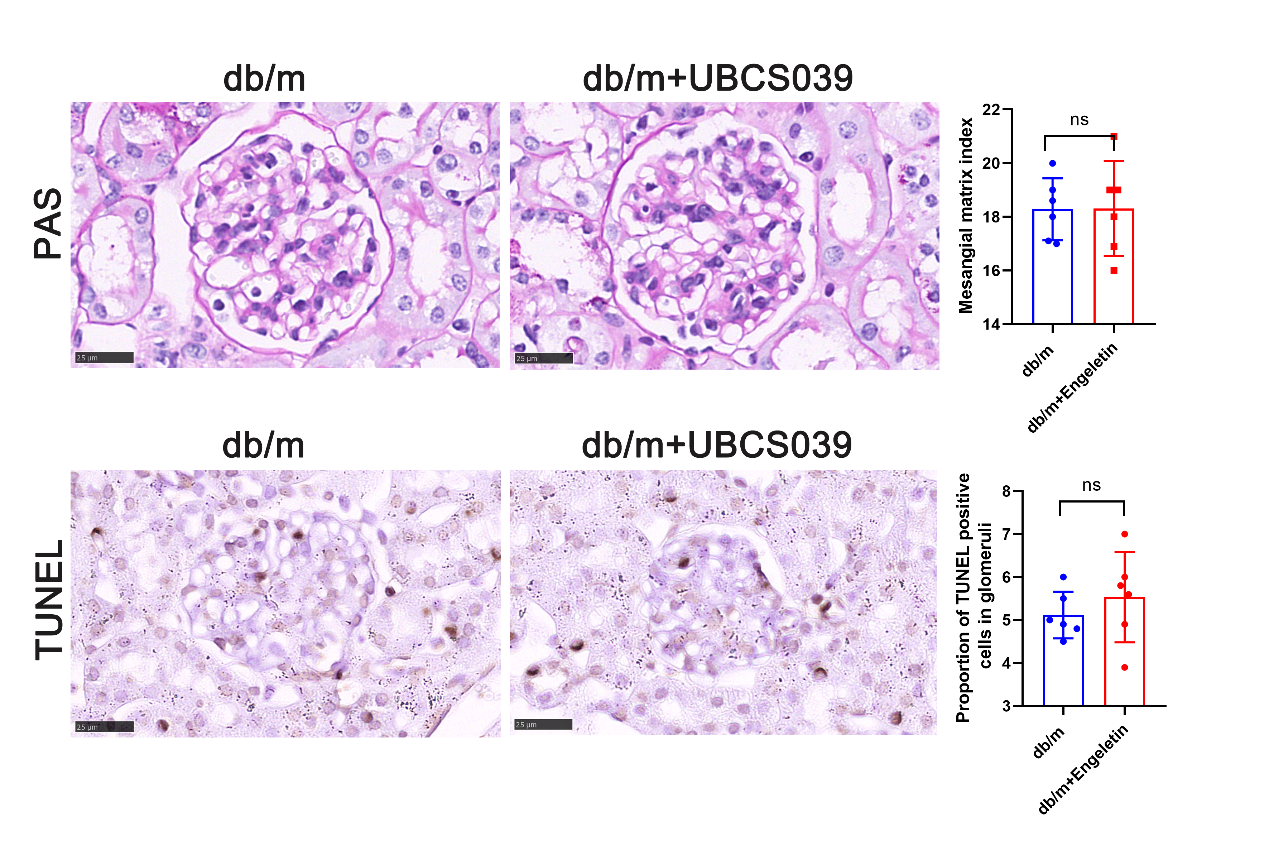

Supplement: Supplemental Material [file IRNF_A_2410396_SM7885.docx]
